# Effects of physiatrist and registered therapist operating acute rehabilitation (PROr) in patients with stroke

**DOI:** 10.1371/journal.pone.0187099

**Published:** 2017-10-26

**Authors:** Tokio Kinoshita, Yukihide Nishimura, Takeshi Nakamura, Takamasa Hashizaki, Daisuke Kojima, Makoto Kawanishi, Hiroyasu Uenishi, Hideki Arakawa, Takahiro Ogawa, Yoshi-ichiro Kamijo, Takashi Kawasaki, Fumihiro Tajima

**Affiliations:** 1 Department of Rehabilitation Medicine, Wakayama Medical University, Wakayama city, Wakayama, Japan; 2 Department of Rehabilitation Medicine, Iwate Medical University, Morioka city, Iwate, Japan; 3 Department of Rehabilitation Medicine, School of Medicine, Yokohama City University, Yokohama city, Kanagawa, Japan; National Cerebral And Cardiovascular Center, JAPAN

## Abstract

**Objective:**

Clinical evidence suggests that early mobilization of patients with acute stroke improves activity of daily living (ADL). The purpose of this study was to compare the utility of the physiatrist and registered therapist operating acute rehabilitation (PROr) applied early or late after acute stroke.

**Subjects and methods:**

This study was prospective cohort study, assessment design. Patients with acute stroke (n = 227) admitted between June 2014 and April 2015 were divided into three groups based on the time of start of PROr: within 24 hours (VEM, n = 47), 24–48 hours (EM, n = 77), and more than 48 hours (OM, n = 103) from stroke onset. All groups were assessed for the number of deaths during hospitalization, and changes in the Glasgow Coma Scale (GCS), National Institute of Health Stroke Scale (NIHSS), and Functional Independence Measure (FIM) at hospital discharge.

**Interventions:**

All patients were assessed by physiatrists, who evaluated the specific needs for rehabilitation, and then referred them to registered physical therapists and occupational therapists to provide early mobilization (longer than one hour per day per patient).

**Results:**

The number of deaths encountered during the PROr period was 13 (out of 227, 5.7%), including 2 (4.3%) in the VEM group. GCS improved significantly during the hospital stay in all three groups, but the improvement on discharge was significantly better in the VEM group compared with the EM and OM groups. FIM improved significantly in the three groups, and the gains in total FIM and motor subscale were significantly greater in the VEM than the other groups.

**Conclusions:**

PROr seems safe and beneficial rehabilitation to improve ADL in patients with acute stroke.

## Introduction

Many stroke guidelines recommend the start of rehabilitation management of acute stroke patients as soon as possible [1–5]. For stroke patients, the start of rehabilitation within 24 hours improves the chance of better outcome of activity of daily living (ADL), quality of life and minimizes costs compared with the start of rehabilitation within 24–48 hours [6–11]. However, in the A Very Early Rehabilitation Trial (AVERT) phase III trial [12], very early and intensive mobilization starting within 24 hours was associated with a significant reduction in the odds of a favorable outcome on the modified Rankin Scale score (mRS) [13,14].

In the AVERT study, physical therapists (PT), occupational therapists (OT), and nurses applied early mobilization in patients with stroke, and the time spent in rehabilitation was 31 (range, 16.5–50.5) min/day/patient [12]. The mobilization program applied at our hospital is different from those described in previous studies that compared the effects of mobilization within 24 hours, 24–48 hours and ≥48 hours [6–12]. At our hospital, all patients are first assessed by the physician, who then refers them to a physiatrist, who in turn evaluates the specific needs for rehabilitation, and then refers them to registered PT and OT to provide early mobilization (longer than one hour per patient). We believe that a well-trained PT and OT can provide intensive and long rehabilitation and early mobilization. Such differences in the rehabilitation programs can possibly result in differences in the risks associated with such programs. In this regard, while previous studies investigated the long-term effect of mobilization within several months from stroke onset, they did not assess the short-term effects of early rehabilitation during acute hospital stay.

At our hospital, it is routine clinical practice for the acute care specialist to request clinical consultation by a physiatrist immediately after the admission of acute stroke patients to the unit. In Japan, physiatrists provide clinical care to more than 4500 new patients of stroke and other disease each year. Based on thorough clinical examination, they select early mobilization tailored to the severity of stroke, type and time since stroke. It is not uncommon for acute stroke patients admitted to the hospital after 11:00 AM to be seen and treatment recommended within 24–48 hours.

In this study, we tested the hypothesis that the physiatrist and registered therapist operating acute rehabilitation (PROr) program used in our hospital is safe and of clinical benefits to patients with acute stroke. To test the hypothesis, we compared the effects of PROr applied within 24, 24–48 and ≥48 hours in acute stroke during short-term hospital stay (2 to 3 weeks).

## Subjects and methods

### Study setting and design

The study was conducted at the Department of Rehabilitation Medicine, Wakayama Medical University Hospital, Wakayama, Japan, between June 2014 and April 2015. The study subjects were patients who presented with neurological deficits at the emergency room, and then diagnosed by neurosurgeons and/or neurologists with stroke and started on treatment. Physiatrists were consulted in the management of all such patients, and recommended immediate rehabilitation therapy.

### Study design

A prospective cohort study, assessment design.

### Subjects

**Fig 1:**
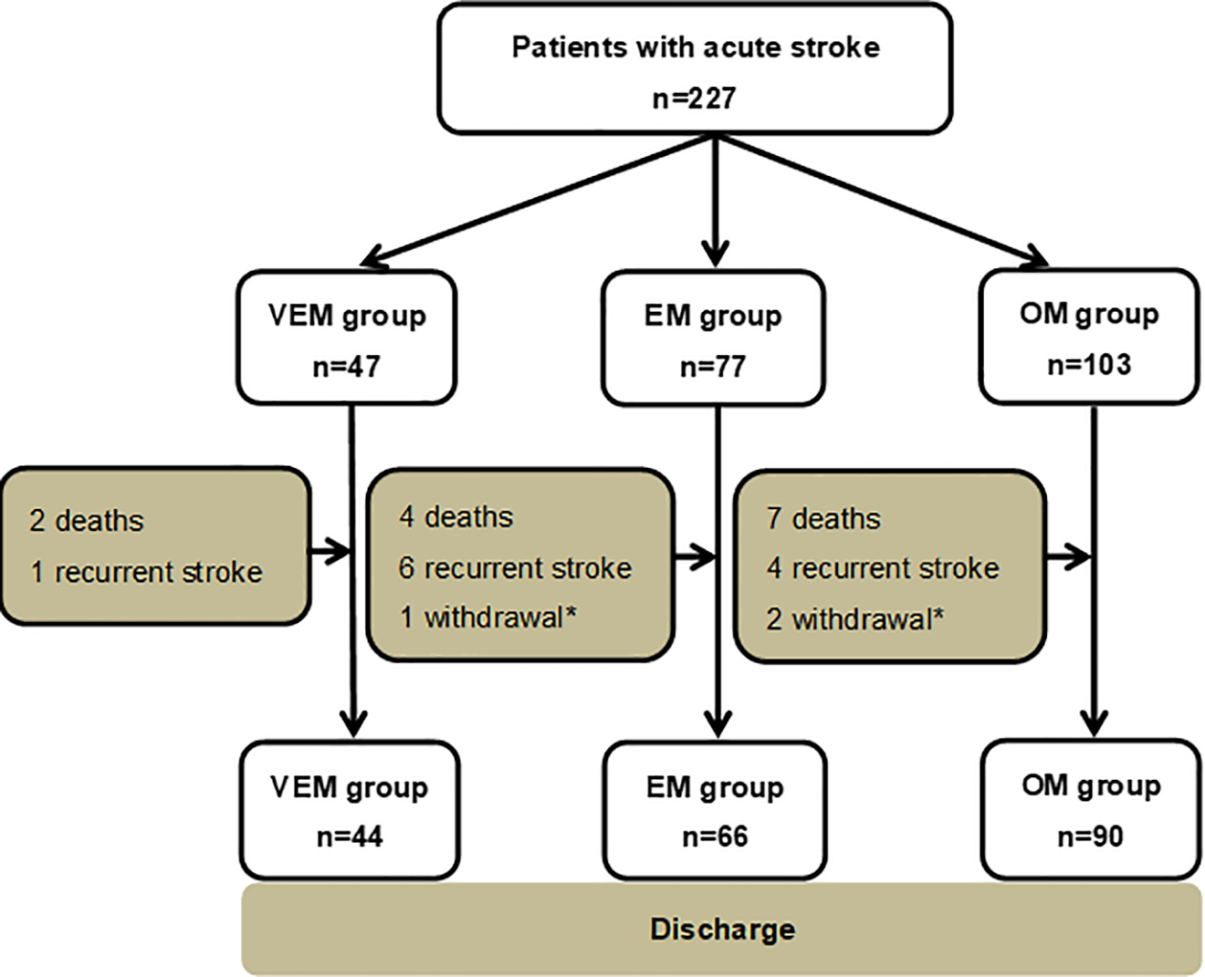
Patients enrolment flow chart. *; Patients with severe heart failure and acute myocardial infarction were excluded from the study. VEM; very early mobilization (started within 24 hours), EM; early mobilization (started 24–48 hours), OM; other mobilization (started ≥48 hours).

A total of 227 patients with acute stroke were included in this study. Only patients aged ≥18 years were included and the following exclusion criteria were applied: premorbid mRS >4 [13,14], concurrent progressive neurologic disorder, severe heart failure, confirmed or suspected lower-limb fracture preventing mobilization, and need for palliative care. In this prospective study, the subjects were divided into three groups based on the time of start of rehabilitation in relation to the onset of stroke. Patients of the first group started the rehabilitation program within 24 hours from stroke onset (Very Early Mobilization; VEM, n = 47), the second group started the program within 24–48 hours (Early Mobilization; EM, n = 77), and the third group started after more than 48 hours (Late Mobilization; OM, n = 103) (Fig 1). The baseline socio-demographic characteristics and stroke details were similar among the three groups. The duration of hospitalization was significantly longer in the OM group than the VEM groups (Table 1).

**Table 1:** Baseline characteristics of patients.

	VEM group	EM group	OM group
N	47	77	103
Age (years)	72.4±2.2	77.3±1.4	77.3±1.2
Gender (females/males)	23 / 24	39 / 38	44 / 59
Height (cm)	159±1.5	158±1.0	158±0.9
Weight (kg)	60±2.1	57±1.3	56±1.2
Type of stroke (Hemorrhage / infarction)	18 / 29	12 / 65	34 / 69
Duration of hospital stay (days)	14.2±1.1 (n = 44)	16.3±1.2 (n = 66)	19.5±1.1^†^ (n = 90)
Time spent in rehabilitation per person (min/day)	73.9±3.3 (n = 44)	69.4±3.0 (n = 66)	69.3±2.4 (n = 90)

Data are mean±SEM.

^†^p<0.05, compared with the VEM group.

VEM; very early mobilization (started within 24 hrs), EM; early mobilization (started within 24–48 hrs), OM; other mobilization (started ≥48 hrs).

### Rehabilitation program (PROr)

PROr was started under medical management of physiatrists. They managed the treatment plan and set the goal for each patient after careful clinical examination. The patients were mobilized out of bed on first rehabilitation. Mobilization, meaning all out of bed activities, was performed by PT and OT. The mobilization on first day was conducted under continuous monitoring of vital signs and consciousness level. Rehabilitation usually involved resistance exercise, exercises of daily living, standing position, and gait training with the long leg orthosis. PROr aims for high intensity and high frequency mobilization as much as possible for each patient. The rehabilitation program was applied 5 days per week.

### Outcome measures

All patients included in this study were assessed on first rehabilitation and discharge. Each measured parameter was evaluated by skilled PT. The outcome measures were the number of deaths and recurrent stroke, Glasgow Coma Scale (GCS), National Institute of Health Stroke Scale (NIHSS), mRS, and Functional Independence Measure (FIM). GCS is a tool used by medical professionals for objective evaluation of the degree of consciousness or coma, and the results are scaled between 3 and 15, with high scores indicating higher levels of consciousness [15,16]. The NIHSS is a 15-item neurological examination stroke scale used to evaluate the effects of acute cerebral infarction on the level of consciousness, language, neglect, visual-field loss, extraocular movement, motor strength, ataxia, dysarthria, and sensory loss [17,18]. The mRS defines six levels of disability [13,14]. FIM is a basic indicator of the severity of disability [19–21]. In this regard, several studies concluded that the FIM is more sensitive for evaluation of ADL than mRS and Barthel Index (BI) [22–26]. FIM (total 18 items) consists of motor subscale (13 items) and cognition subscale (5 items), each of which is assessed against a seven-point ordinal scale.

### Ethical considerations

The study protocol was approved by the ethics review committee of Wakayama Medical University and conformed to the Declaration of Helsinki. All patients or their close relatives provided consent to the rehabilitation treatment.

### Statistical analysis

All data were expressed as mean ± standard error of the mean (SEM). Changes in GCS, NIHSS and FIM from values measured before the first rehabilitation to that on discharge were included in the present analysis. All data, except GCS, NIHSS, mRS and FIM, were tested by one-way analysis of variance. Subsequent posthoc tests to compare the difference among the three groups (VEM, EM, and OM) were performed by Tukey-Kramer test. Data of GCS, NIHSS, mRS and FIM were tested by Kruskal-Wallis test. We used the Dunn's test for subsequent post-hoc test to determine the significance of differences among the three groups. Differences between before and after rehabilitation were examined by using the Wilcoxon signed-rank test. The χ2 test was used to compare the number of deaths and recurrent stroke among the groups. Differences were considered statistically significant at P level of <0.05. All statistical evaluations were performed by using Graph Pad Prism 6 software (GraphPad Software Inc, CA).

## Results

### Mortality and recurrent stroke

The total number of deaths was 13 (out of 227, 5.7%) and 11 (4.8%) developed another stroke during the study period. The numbers of deaths were 2 (4.3%), 4 (5.2%) and 7 (6.8%) in the VEM, EM and OM groups, while the respective numbers of repeat stroke were 1 (2.1%), 6 (7.8%), and 4 (3.9%). The numbers of deaths and repeat stroke were not significantly different among the three groups.

### Glasgow coma scale

The GCS improved significantly in the VEM (14.7±0.1), EM (13.7±0.3) and OM (13.8±0.3) groups at discharge (range: 3–62 days) compared with the respective values before the first rehabilitation (13.8±0.3, 13.0±0.4 and 12.6±0.4). Further analysis showed that the GCS of the VEM at discharge was significantly higher than that of the EM and OM groups (Table 2), but there was no significant difference in the gain of GCS (VEM; 0.9±0.2, EM; 0.7±0.2, OM; 1.2±0.2) among the three groups.

**Table 2:** Changes in Glasgow Coma Scale (GCS), National Institute of Health Stroke Scale (NIHSS), modified Rankin Scale (mRS) and Functional Independence Measure (FIM).

	First rehabilitation			Discharge		
	VEM n = 44	EM n = 66	OM n = 90	VEM n = 44	EM n = 66	OM n = 90
Glasgow Coma Scale	13.8±0.3	13.0±0.4	12.6±0.4	14.7±0.1^†^	13.7±0.3^†^^,^*	13.8±0.3^†^^,^*
NIHSS	7.3±1.1	10.6±1.3	12.2±1.2	4.4±1.0^†^	8.1±1.1^†^	8.3±1.0^†^^,^*
mRS	4.0±0.2	4.1±0.2	4.3±0.1	3.0±0.2^†^	3.5±0.2^†^	3.6±0.1^†^
Total FIM	53.4±3.9	54.3±4.2	51.3±3.5	86.0±5.1^†^	74.3±4.7^†^	71.2±3.9^†^
Motor subscale	30.3±2.8	33.3±2.9	31.7±2.4	58.8±4.0^†^	50.9±3.5^†^	47.6±3.0^†^
Cognition subscale	23.1±1.6	21.0±1.5	19.6±1.3	27.3±1.3^†^	23.4±1.4^†^	23.6±1.2^†^

Data are mean±SEM.

^†^p<0.05, compared with first rehabilitation and discharge.

*p<0.05, compared with the VEM group.

See Table 1 for the definition of the three groups.

### National Institutes of Health Stroke Scale

NIHSS improved significantly in the VEM (4.4±1.0), EM (8.1±1.1) and OM (8.3±1.0) groups at discharge compared with the respective values before the first rehabilitation (7.3±1.1, 10.6±1.3, and 12.2±1.2). Further analysis showed that NIHSS of the VEM group at discharge was significantly lower than that of the OM group (Table 2), but there was no significant difference in the gain of NIHSS (VEM; -2.9±0.5, EM; -2.5±0.4 and OM; -3.9±0.5) among the three groups.

### Modified Rankin Scale

The mRS improved significantly in the VEM (3.0±0.2), EM (3.5±0.2) and OM (3.6±0.1) groups at discharge compared with the respective values before the first rehabilitation (4.0±0.2, 4.1±0.2 and 4.3±0.1) (Table 2). However, there was no significant difference in the gain of mRS (VEM; -1.0±0.1, EM; -0.5±0.1 and OM; -0.7±0.1) among the three groups.

### Functional Independence Measure

**Fig 2:**
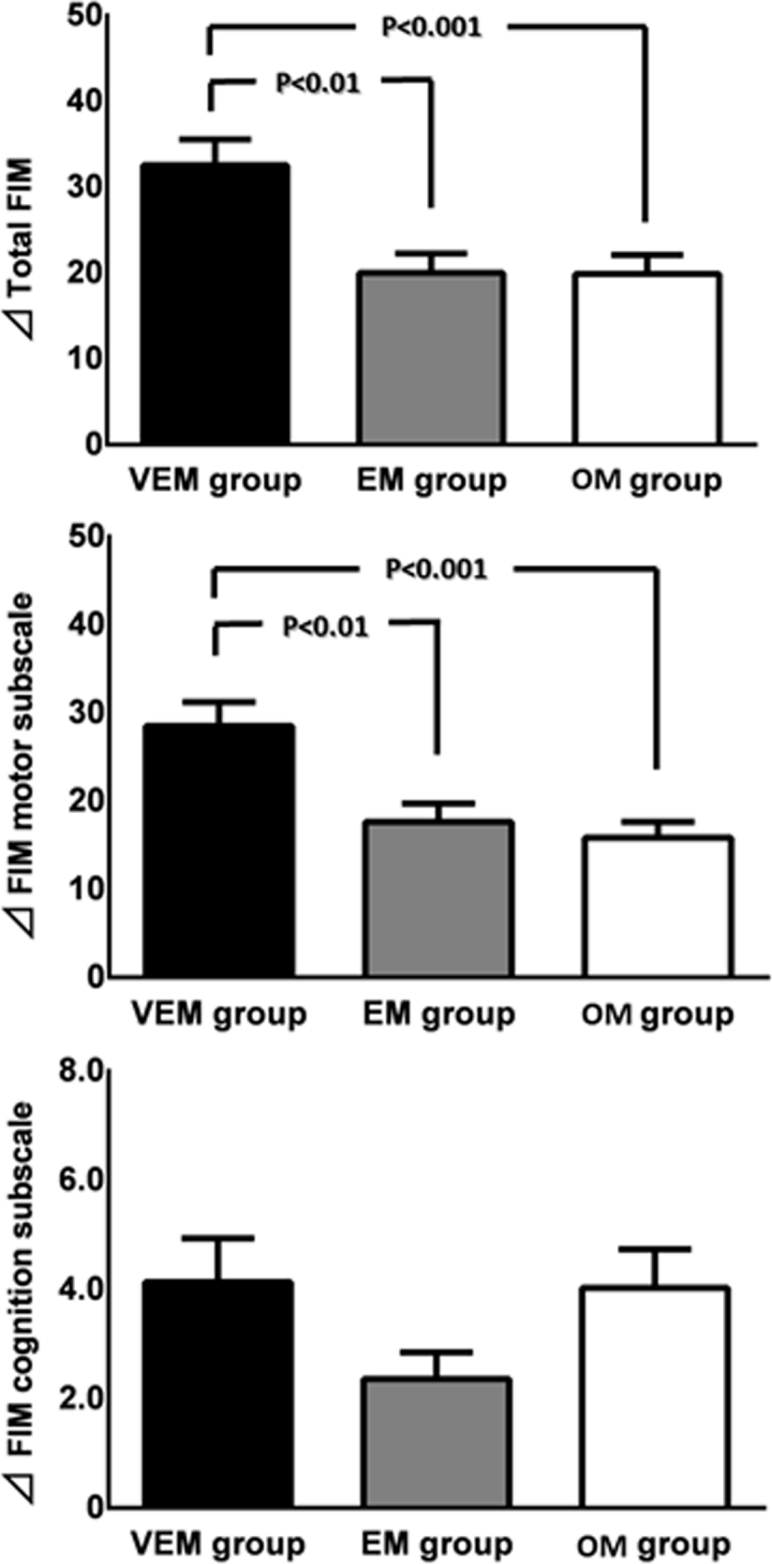
Gain in Functional Independence Measure (FIM). Data are mean±SEM. See Table 1 for the definition of the three groups.

Total FIM improved significantly in the VEM (86.0±5.1), EM (74.3±4.7) and OM (71.2±3.9) groups at discharge compared with the respective values before the first rehabilitation (53.4±3.9, 54.3±4.2 and 51.3±3.5) (Table 2). Also, the motor and cognition subscales of FIM were significantly improved, compared with the value before the first rehabilitation (Table 2). The gains in the total FIM (32.6±3.0) and motor subscale (28.5±2.7) in the FIM group were significantly greater than those in the EM (20.2±2.3, 17.7±2.1, respectively) and OM (19.9±2.2, 15.9±1.8, respectively) groups. However, the gain in the cognition subscale of FIM (VEM; 4.1±0.8, EM; 2.7±0.5 and OM; 4.0±0.7) was not significantly different among the three groups (Fig 2).

## Discussion

The followings were the major findings of the present study; *1)* there were no significant differences among the three groups with regard to the number of deaths and recurrent stroke, *2)* the GCS at discharge showed a significantly better improvement in the VEM group compared with the EM and OM groups, *3)* the gains in total FIM and motor subscale during hospitalization showed significantly higher improvement in the VEM than EM and OM groups. *4)* However, the improvements in the mRS and gain of GCS was not significantly different among the three groups. These findings suggest that PROr poses no risk and can improve FIM during acute phase stroke.

In the present study, PROr was started immediately after stroke under the clinical management of physiatrists, emergency physicians and neurologists. Patients were mobilized out of bed from the first rehabilitation. Mobilization, meaning all out of bed activities, was conducted under the supervision of our PT and OT specialists. Mobilization on the first day was performed while monitoring vital signs and consciousness level. PROr usually involves resistance exercise, cardiopulmonary exercise, exercises of daily living, standing position, and gait training with long leg orthosis. In the AVERT phase III trial, early mobilization was conducted for about 30 minutes per person by the therapist or/and nurse [12]. In comparison, the PROr applied in the present study was about 70 minutes per person. We believe that the main reason for the successful gain in FIM was that the 70-min PROr was performed by well-trained PT and OT under guidance of the physiatrist.

The AVERT phase II trial described no significant difference in the number of deaths between intensive mobilization that started within 24 hours and mobilization that started 24–48 hours after stroke onset [6]. Furthermore, Greening et al. [27] reported that early rehabilitation (started within 48 hours of stroke onset) during hospital admission for chronic respiratory disease neither reduced the risk of subsequent readmission nor enhanced recovery of physical function and mortality. In the present study, the timing of the start of rehabilitation relative to the onset of stroke did not significantly alter the number of deaths and recurrent stroke. Our results add support to the findings of the AVERT phase II trial [6]. In the AVERT phase II trial, the death rate was 8.5% (6/71) when rehabilitation started two weeks within stroke onset and 13.2% (5/38) for within 24 hours [6]. In our study, the death rate was 5.7% when rehabilitation started about two weeks and 4.3% in the VEM group. Considered together, the above studies and the present findings support the view that PROr is clinically beneficial and is not associated with worsened mortality.

Momozaki et al. [28] reported that the provision of rehabilitative care by board-certificated physiatrists correlated with improved functional recovery of elderly patients with hip fracture after rehabilitation. In the present study, physiatrists examined the patients and recommended the treatment. We believe that the main reason for the benefits observed in this study was that the physiatrists managed the treatment plans and set treatment goals tailored to the needs of the individual patient, which in most cases was independent of the management by other clinical specialties. In this regard, the physiatrists well understand the physiological effects of standing and exercise, in addition to the pathophysiological functions, and they can rule out the risks of cardiovascular and respiratory systems, as well as vertebral, bone and joint problems. Even if the patients were in coma and/or under mechanical ventilation, PT and OT applied early mobilization based on the advice provided by the physiatrist.

In the present study, although PROr had no effect on mRS, there was a significant improvement in FIM in the 24 hours group compared with the EM and OM groups. There is general agreement that FIM is a basic indicator of the severity of disability [19–21]. Furthermore, several studies concluded that the FIM is more sensitive for evaluation of ADL than mRS and BI [13–2].

The total FIM and motor subscale of FIM increased in all three groups. However, the gain was significantly greater when mobilization was applied within the first 24 hour than later (VEM group versus the EM and OM groups). Furthermore, the cognitive subscale of the FIM also increased in all three groups, though no significant difference was found among the three groups. Moriki et al. [29] described that the sitting position improved the GCS score in patients with cerebral disorders and disturbances of consciousness. In our study, all patients were mobilized out of bed on the first rehabilitation and the cognitive subscale of the FIM improved in all three groups.

The present study has certain limitations. First, the study was a prospective cohort study, not a randomized controlled trial. Second, although the results showed a better functional outcome in patients who started mobilization within the 24 hours, we cannot make a definitive claim about the impact of time of rehabilitation in patients with acute stroke. However, our study extends the message that patients with stroke seem to benefit from rehabilitation applied within 24 hours of stroke onset.

## Conclusions

PROr was effective in improving total FIM in patients with stroke when it was started within 24 hours from onset of stroke, and was not associated with serious complications or side effects.

## Supporting information

S1 File Raw data of the present study. This file is raw data of Glasgow Coma Scale, National Institute of Health Stroke Scale, modified Rankin Scale (mRS) and Functional Independence Measure.
(XLSX)
